# The E3 ubiquitin-protein ligase Nedd4-2 regulates the sodium chloride cotransporter NCC but is not required for a potassium-induced reduction of NCC expression

**DOI:** 10.3389/fphys.2022.971251

**Published:** 2022-09-07

**Authors:** Lena L. Rosenbaek, Federica Petrillo, Miguel X. van Bemmelen, Olivier Staub, Sathish K. Murali, Robert A. Fenton

**Affiliations:** ^1^ Department of Biomedicine, Aarhus University, Aarhus, Denmark; ^2^ Leducq Foundation Potassium in Hypertension International Network, Massachusetts, United States; ^3^ Department of Pharmacology and Toxicology, University of Lausanne, Lausanne, Switzerland; ^4^ Department of Biomedical Sciences, University of Veterinary Medicine, Vienna, Austria

**Keywords:** E3 ligase, ubiquitin, ubiquitylation, co-transporter, aldosterone, potassium

## Abstract

Na^+^ and K^+^ balance is influenced by the activity of the sodium chloride cotransporter NCC in the distal convoluted tubule. NCC activity and abundance are reduced by high extracellular K^+^. The E3 ubiquitin ligase neural precursor cell expressed developmentally downregulated 4–2 (Nedd4-2) has been proposed as a modulator of NCC abundance. Here, we examined the functional role of Nedd4-2 on NCC regulation and whether Nedd4-2 is important for the effects of high extracellular K^+^ on NCC. Total and plasma membrane levels of ubiquitylated NCC were lower in NCC-expressing MDCKI cells after Nedd4-2 deletion. NCC and phosphorylated NCC (pT58-NCC) levels were higher after Nedd4-2 deletion, and NCC levels on the plasma membrane were elevated. No significant changes were seen after Nedd4-2 knockdown in the levels of SPAK and phosphorylated SPAK (pS373-SPAK), the major NCC regulatory kinase. Nedd4-2 deficiency had no effect on the internalization rate of NCC from the plasma membrane, but NCC protein half-life was increased. In *ex vivo* experiments with kidney tubule suspensions from Nedd4-2 knockout (KO) mice, high K^+^ reduced total and pT58-NCC regardless of genotype. We conclude that Nedd4-2 is involved in ubiquitylation of NCC and modulating its plasma membrane levels and degradation. However, Nedd4-2 does not appear to be important for K^+^ induced reductions in NCC abundance.

## Introduction

In the kidney distal convoluted tubule (DCT), the apical thiazide-sensitive sodium chloride cotransporter (NCC) plays an important role in determining the urinary excretion of Na^+^, K^+^, Mg^2+^, and Ca^2+^ and in maintaining blood pressure (Subramanya and Ellison, 2014; Hoorn et al., 2020). The importance of NCC is apparent in patients with Gitelman’s syndrome, caused by inactivating mutations in the NCC-encoding *SLC12A3* gene, whom usually present with hypotension, hypokalemia, hypomagnesemia, metabolic alkalosis, and hypocalciuria (Mastroianni et al., 1996; Simon et al., 1996; Lemmink et al., 1998; Monkawa et al., 2000). Overactivation of NCC, due to genetic mutations in upstream regulatory kinases or E3 ubiquitin ligases, is the basis of hypertension in patients with Gordon syndrome (pseudohypoaldosteronism type II, PHAII) (Hadchouel et al., 2016). NCC is regulated, directly or indirectly, in response to various hormones, including aldosterone, angiotensin II and vasopressin. In addition, alterations in extracellular fluid (ECF) K^+^ concentrations, for example after alterations in dietary K^+^ intake, also modulate NCC activity (Hoorn et al., 2020). Low dietary K^+^ intake increases NCC phosphorylation [surrogate marker of NCC activity (Hadchouel et al., 2016)] and abundance, whereas high dietary K^+^ intake inhibits NCC (Frindt and Palmer, 2010; Sorensen et al., 2013; van der Lubbe et al., 2013; Rengarajan et al., 2014; Terker et al., 2015). The lower NCC phosphorylation following high dietary K^+^ intake is due to reduced activity of the inwardly-rectifying potassium channels Kir4.1/Kir5.1 (a heterotetramer), changes in the basolateral plasma membrane potential, and reduced activity of the WNK-SPAK kinase signaling pathway (Piala et al., 2014; Bazua-Valenti et al., 2015; Cuevas et al., 2017; Wu et al., 2019). Altered dietary K^+^ also results in changes in activity of the Kelch-like protein 3 (KLHL3) and Cullin 3 (Cul3) E3 ubiquitin ligase complex that targets WNK kinases for degradation, altering NCC activity (Ishizawa et al., 2016; Cheng et al., 2019; Kortenoeven et al., 2021; Murali et al., 2021; Poulsen et al., 2021).

In addition to phosphorylation, NCC is regulated by ubiquitylation and subsequent degradation (Arroyo et al., 2011; Rosenbaek et al., 2017a), with accumulating evidence for the involvement of the E3 ligase ubiquitin-protein ligase neuronal precursor cell developmentally downregulated 4–2 (Nedd4-2, also known as Nedd4L). Nedd4-2 belongs to the Nedd4 family of ubiquitin ligases characterized by an amino terminal Ca^2+^ phospholipid binding (C2) domain, 4 WW protein-protein interaction domains, and a carboxyl terminal HECT-type ubiquitin ligase domain. NCC abundance and blood pressure are higher in renal tubule-specific Nedd4-2 knockout mice (Arroyo et al., 2011; Ronzaud et al., 2013), whereas NCC levels are lower in mice lacking the Nedd4-2 regulatory protein serum-glucocorticoid-regulated kinase 1 (Sgk1) (Arroyo et al., 2011; Faresse et al., 2012). NCC levels are also lower in mice after dietary Mg^2+^ restriction, but these effects are not observed in Nedd4-2 knockout mice (Ferdaus et al., 2019). Although direct effects of Nedd4-2 on NCC are supported by studies in cultured cells (Arroyo et al., 2011), other studies have suggested they may be indirect due to alterations in the WNK-SPAK kinase cascade (Roy et al., 2015; Furusho et al., 2020; Hoorn et al., 2020). For example, it was recently shown that Kir4.1 levels are higher in kidney-specific Nedd4-2 knockout mice, which would activate the WNK-SPAK pathway and increase phosphorylated NCC levels (Wu et al., 2020).

To investigate further the role of Nedd4-2 in modulation of NCC under normal conditions or during alterations in ECF K^+^ concentrations we assessed NCC function in kidney cells lacking Nedd4-2 *in vitro*, and in *ex vivo* kidney tubules from Nedd4-2 knockout (KO) mice. Results from our study indicate that NCC and Nedd4-2 directly interact resulting in increased NCC ubiquitylation, decreased NCC plasma membrane abundance, and reduced NCC protein half-life. However, the ability of high K^+^ to reduce NCC levels is still apparent in the absence of Nedd4-2, suggesting Nedd4-2 activity is not required for this mechanism.

## Experimental procedures

### Yeast two hybrid (Y2H) assay

Y2H assays were performed by Hybrigenics® using the carboxyl-terminus (amino acids 606–1021) of human NCC as bait and protein fragments from an adult mouse kidney library as prey. Both N-LexA-bait-C and N-Gal4-bait-C fusion vectors were used and a total of 121.5 million interactions were analyzed.

### Generation of Nedd4-2 MDCKI human NCC (hNCC) knockdown (KD) cell lines

MDCKI-hNCC cells were cultured as previously described in a 24 well plastic plate until 70–85% confluency (Rosenbaek et al., 2017b). Cells were transduced with custom-made MISSION lentiviral transduction particles (Sigma) targeting Nedd4-2 (XP_013964260) in the HECT domain (shRNA sequence: CTG​TGG​ATC​GAA​TTT​GAA​TCA). Non-mammalian scrambled shRNA transduction particles (SHC002V) were used as control. To enhance transduction, cells were treated with 8 µg/ml hexadimethrine bromide in complete media (DMEM, high glucose, GlutaMAX, pyruvate (ThermoFisher #31966021) with 10% DBS), prior to addition of the lentiviral particles (multiplicity of infection of 1.5). Transduced cells were selected by the addition of 4 µg/ml puromycin in complete media after 24 h and colonies allowed to develop. Multiple clonal cell lines were isolated and cultured with 4 µg/ml puromycin in complete media. Lines were characterized by examination of cell morphology, growth characteristics, and Nedd4-2 protein and mRNA expression by western blotting and quantitative reverse transcriptase PCR (RT-qPCR), respectively. RT-qPCR was performed as described (Rosenbaek et al., 2017b), using Nedd4-2, NCC, or Nedd4 specific primers (Nedd4-2; forward: 5′-CAC​TGG​AGG​GTG​CCA​AGG​AT-3′, reverse: 5′-CCG​TTG​GGC​GCT​ATC​CTC​AT-3’. NCC; forward: 5′- ACA​GAG​TCA​AGT​CCC​TTC​GG-3′, reverse: 5′- GGA​TGA​CTG​GAG​GTC​TGA​GG-3’. Nedd4; forward: 5′- CTC​ACT​GTG​TGC​TAC​CGG​AC-3′, reverse: 5′- ATG​CTG​TTA​GGG​TCG​ATC​TCA​CT-3′). Primers against 18S-rRNA were used for normalization (forward: 5′-GGA​TCC​ATT​GGA​GGG​CAA​GT-3′, reverse: 5′-ACG​AGC​TTT​TTA​ACT​GCA​GCA​A-3′).

### Cell surface biotinylation coupled with immunoprecipitation (IP)

Cell lines were seeded at a density of 3 x 10^5^ cells/cm^2^ on semipermeable transwell plates (Corning #3450 or Greiner #657641) coated with basement membrane extract (BME) (Cultrex® Basement Membrane Extract, PathClear, R&D Systems) and grown in complete media until confluent. Cells were induced with 10 μg/ml tetracycline HCl (Sigma) for 16–20 h at 37 °C prior to experiment. Cells were washed twice in ice-cold PBS-CM (PBS, 1 mM CaCl2, 0.1 mM MgCl2, pH 7.5) and apical membrane proteins were biotinylated with 1.0 mg/ml of sulfosuccinimidyl 2-(biotin-amido)-ethyl-1,3-dithiopropionate (EZ-link Sulfo-NHS-SS-biotin, Invitrogen) in ice-cold biotinylation buffer (10 mM triethanolamine, 2 mM CaCl2, 125 mM NaCl, pH 8.9) for 30 min at 4 °C with mild agitation. Cells were washed twice with quenching solution (50 mM Tris-HCl in PBS-CM, pH 8) and once in PBS-CM to remove the excess of biotin, before being lysed in lysis buffer (50 mM Tris-HCl, 150 mM NaCl, 1% Triton X-100, 5 mM EDTA (pH 7.5), 20 mM N-ethylmaleimide (Sigma), 22 μM PR619 (Abcam), 5 μg/ml leupeptin, 100 μg/ml Pefabloc, and PhosSTOP phosphatase inhibitor tablets (Roche Diagnostics). Lysates were sonicated and centrifuged at 10,000 × g for 5 min at 4°C. One fraction of the supernatant was retained for total NCC protein estimation (total fraction). The remaining was incubated for 1 h in spin columns with 200 μl of Neutravidin Plus UltraLink Resin (Invitrogen) under rotation at room temperature (RT). Neutravidin beads were washed in PBS (pH 7.2) with inhibitors and biotinylated proteins eluted with 50 mM DTT in IP lysis buffer (20 mM Tris-Base, 135 mM NaCl, 1% NP-40, 5 mM EDTA, pH 7.4) for 1 h at RT with rotation. One fraction of the eluted sample was retained for surface protein estimates (biotinylated fraction). 180 µl of the remaining sample was subjected to immunoprecipitation (IP) using 40 µl FLAG-M2 affinity beads (A2220, Merck) at 4 °C with rotation overnight. Beads were washed extensively in IP lysis buffer with inhibitors and eluted using 200 µg/ml FLAG-peptide (DYKDDDDK, Genscript) in TBS buffer (10 mM Tris-HCl, 150 mM NaCl, pH 7.4). Laemmli sample buffer with a final DTT concentration of 100 mM was added to all fractions, which were subsequently heated for 15 min at 60°C. Data was obtained from three independent experiments with three replicates from each cell line per experiment.

### Immunoprecipitation of total cell lysates

Cells were cultured in T25 plastic flasks and grown in complete media until confluent. Cells were induced with 10 μg/ml tetracycline HCl for 16–20 h at 37°C prior to experiment. Cells were washed twice in ice-cold PBS-CM (PBS, 1 mM CaCl2, 0.1 mM MgCl2, pH 7.5) and lysed by addition of 500 µl IP lysis buffer. After sonication and centrifugation (10,000 × g for 5 min at 4°C), one fraction of the supernatant was retained for total NCC protein estimation (total fraction). The remaining lysate was subjected to IP with FLAG-M2 affinity beads as above.

### Biotin-based internalization assay

The assay was performed as previously described (Rosenbaek et al., 2014). Data was obtained from three independent experiments with three replicates for each time point per experiment.

### NCC protein half-life assay

Cell lines were seeded on semipermeable transwell plates and grown in complete media until confluent. Cells were induced with 10 μg/ml tetracycline HCl for 16–20 h at 37°C prior to experiment. Cells were washed once with complete media and then incubated with 100 μg/ml (355 µM) cycloheximide (Sigma) and 5 μM Actinomycin D (Sigma) in DMEM GlutaMAX for up to 10 h. Cells were washed in PBS-CM and lysed in Laemmli sample buffer containing 100 mM DTT. Samples were sonicated, heated at 60 °C for 15 min and subjected to immunoblotting. For calculation of the protein half-life, average band densities for each time point were normalized to time zero and fitted using nonlinear regression and a one-phase exponential decay equation using GraphPad Prism software. Data was obtained from 8 independent experiments with two replicates for each individual timepoint per experiment.

### Ex vivo kidney tubule suspensions

All experimental procedures were approved by the Swiss Federal Veterinary Office and carried out in accordance with the local animal welfare act. Renal tubule-specific Nedd4L^flox/flox^/Pax8-rTA/LC1 (Nedd4L-Pax8/LC1) KO mice (Ronzaud et al., 2013) were generated by supplementing the drinking water for eleven days with 2 mg/ml doxycycline and 20 mg/ml sucrose. Single transgenic homozygous Nedd4L^flox/flox^-Pax8 or Nedd4L^flox/flox^-LC1 littermates were used as controls. Animals were 8–9 weeks of age at the onset of the treatment and were used for the experiments two weeks after switching to plain drinking water. Mouse kidney tubules were subsequently isolated from the mice as described previously (Poulsen et al., 2021) with modifications. Briefly, mice were anesthetized with isoflurane and the left ventricle was perfused using 10 ml of isolation buffer (140 mM NaCl, 0.4 mM KH_2_PO_4_, 1.6 mM K_2_HPO_4_, 1 mM MgSO_4_, 10 mM Na-Acetate, 1 mM α-ketoglutarate, 1.3 mM calcium-gluconate, 30 mM glucose, 2mg/ml collagenase II, 0.376 mg/ml glycine, 0.05 mg/ml DNase I and 0.05mg/ml trypsin inhibitor). Kidneys were isolated, their capsules removed, the kidneys chopped into small pieces using a scalpel and tissue placed into 2 ml of pre-warmed isolation buffer. Tissue was digested at 37°C for 5 min with shaking after which larger pieces of non-digested tissue were settled at the bottom of the tube. The supernatant containing tubules was transferred into a new tube, fresh isolation buffer added, and tissue gently disrupted using plastic Pasteur pipettes. Tissue was incubated at 37°C for an additional 5 min before the supernatant was transferred to a new tube and washed 3 times with incubation media (1.8 mM CaCl_2_, 0.81 mM MgSO_4_, 44 mM NaHCO_3_, 110 mM NaCl, 0.9 mM NaH_2_PO_4_, 0.1 mg/L Ferric nitrate, 0.4 mM glycine, 4 mM l-glutamine, 25 mM d-glucose, 1 mM sodium pyruvate and 0.5 mM KCl). Isolated tubules were resuspended in 20 ml of incubation media and 1 ml of the suspension was added to each well of a 12-well plate. Isolated tubules were incubated at 37°C for 2 h after which the incubation media was gently removed from each well and tubules were incubated with new media containing either 0.5 mM, 3.5 mM, or 8 mM KCl at 37°C. Appropriate concentrations of choline chloride were used to equalize the chloride concentration between different media. After 24 h the media was gently removed and Laemmli sample buffer containing 15 mg/mL DTT was added to each well. Samples were sonicated and denatured at 60°C for 15 min before storage at 4°C. SDS-PAGE and coomassie staining were used to normalize protein loading of samples prior to immunoblotting. Data was obtained from 4-5 independent experiments with 4 replicates for each individual condition per experiment.

### Immunoblotting and antibodies

Protein samples were separated using 4–15% gradient polyacrylamide gels (Criterion TGX Precast Protein Gels, BioRad) and transferred electrophoretically to PVDF membranes. Immunoblotting was performed using standard methods and the following primary antibodies: rabbit polyclonal antibodies against NCC (SPC-402D, StressMarq), phosphorylated Threonine-58 NCC (pT58-NCC) (Pedersen et al., 2010), Nedd4-2 (Kamynina et al., 2001), phosphorylated Serine373 SPAK/Serine325 OSR1 (pS373-SPAK) (07–2273, Sigma), SPAK/OSR1 (Moriguchi et al., 2005), Kir4.1 (APC-035, Alomone), Kir5.1 (LS-C177333, LS Bio), Proteasome 20S (ab3325, Abcam) and Actin (A2066, Sigma) plus a mouse monoclonal NCC antibody (Kortenoeven et al., 2021)**.** Blots were developed using SuperSignal West Femto chemiluminescent substrate (Thermo Scientific, Denmark) or Amersham ECL Western Blotting Detection Reagent (GE Healthcare) detection and the intensity of the bands were quantified using Image Studio Lite (Qiagen) densitometry analysis.

### Statistical analysis

All data are shown as mean ± S.E.M. For the in vitro studies, the two groups were assessed using Student t-test. For internalization and *ex vivo* studies two-way ANOVA followed by the Tukey’s multiple comparison test was used. Significance was considered at *p* < 0.05. Individual sample sizes (n) are described in the respective figure legends.

## Results

### Nedd4-2 interacts with NCC

**FIGURE 1:**
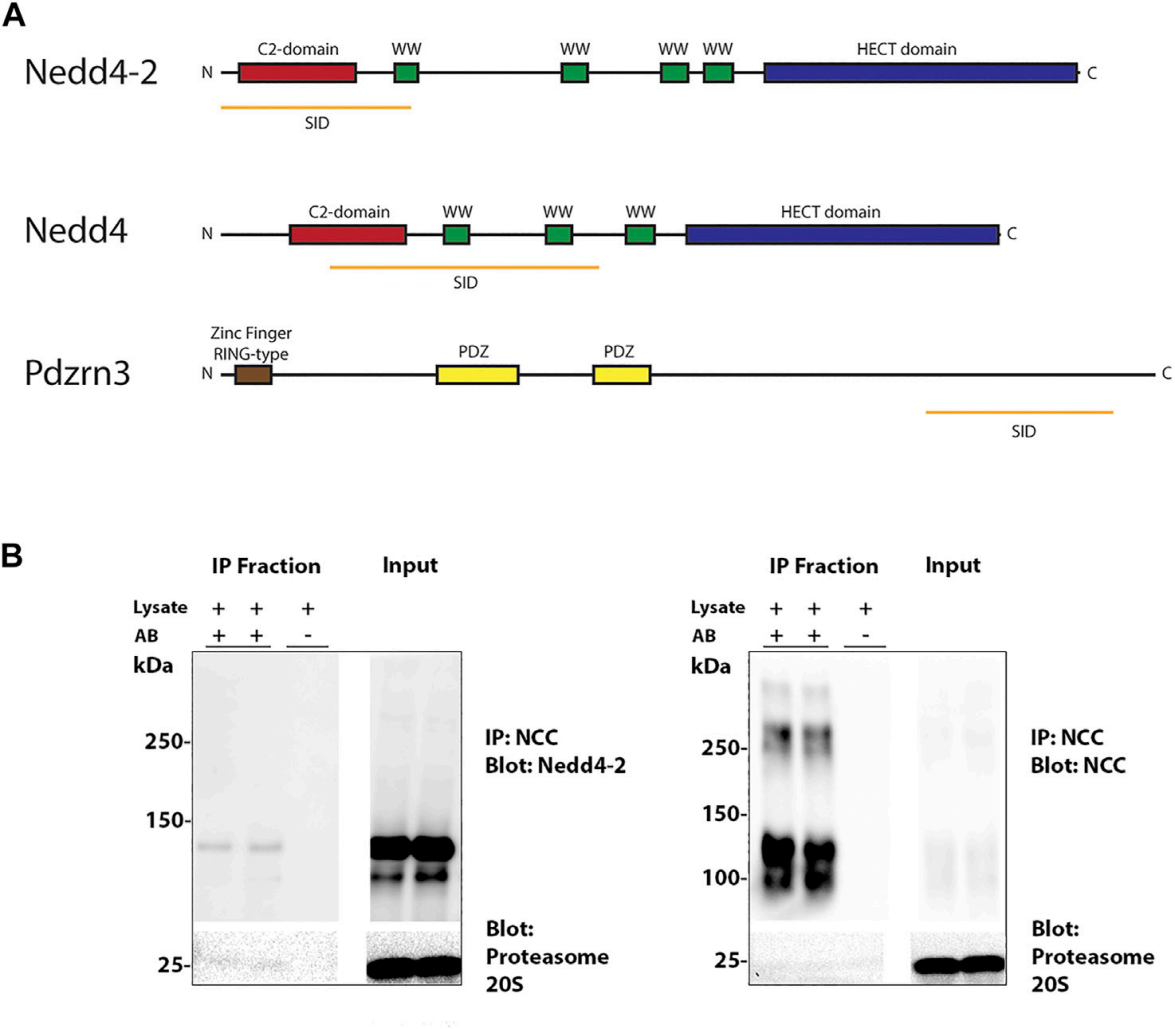
Nedd4-2 interacts with NCC. **(A)** Three E3 ubiquitin ligases were found in a Y2H assay with the C-terminus of NCC as bait. Nedd4-2, Nedd4, and Pdzrn3. The orange line represents the Selected Interaction Domain (SID), corresponding to the amino acid sequence shared by all prey fragments matching the same reference protein. **(B)** NCC immunoprecipitation (IP) of Nedd4-2 from MDCKI-hNCC cells. IP was performed using FLAG-M2 affinity beads and negative controls were performed in the absence of antibody **(A,B)**.

The carboxyl terminus intracellular tail of NCC is highly ubiquitylated on minimally 12 sites in human or mouse (Arroyo et al., 2011; Rosenbaek et al., 2017a). In an attempt to prove direct interaction of NCC and Nedd4-2 and identify other potential E3 ubiquitin-protein ligases that may be important for NCC ubiquitylation, Y2H assays were performed using a bait fragment corresponding to amino acid 606–1021 of human NCC and a mouse kidney library. 16 proteins were identified (Supplementary Table S1), including Nedd4-2, and the E3 ubiquitin-protein ligases Nedd4 and the PDZ Domain Containing Ring Finger 3 (Pdzrn3). The selected interacting domain (SID) of Nedd4-2 and Nedd4 covers the C2-domain and one or two of the WW-domains, respectively, whereas the SID of Pdzrn3 does not cover any identified structural or functional domains (Figure 1A). To verify the interaction between NCC and Nedd4-2, NCC was co-immunoprecipitated from polarized MDCKI cells with tetracycline inducible hNCC expression (Rosenbaek et al., 2017b) and protein lysates probed with a Nedd4-2 antibody. Nedd4-2 was present in NCC positive co-immunoprecipitated samples, but not in negative controls performed in the absence of the immunoprecipitating flag-tag antibody (Figure 1B).

### Generation of MDCKI-hNCC cell lines with Nedd4-2 knock down (KD)

**FIGURE 2:**
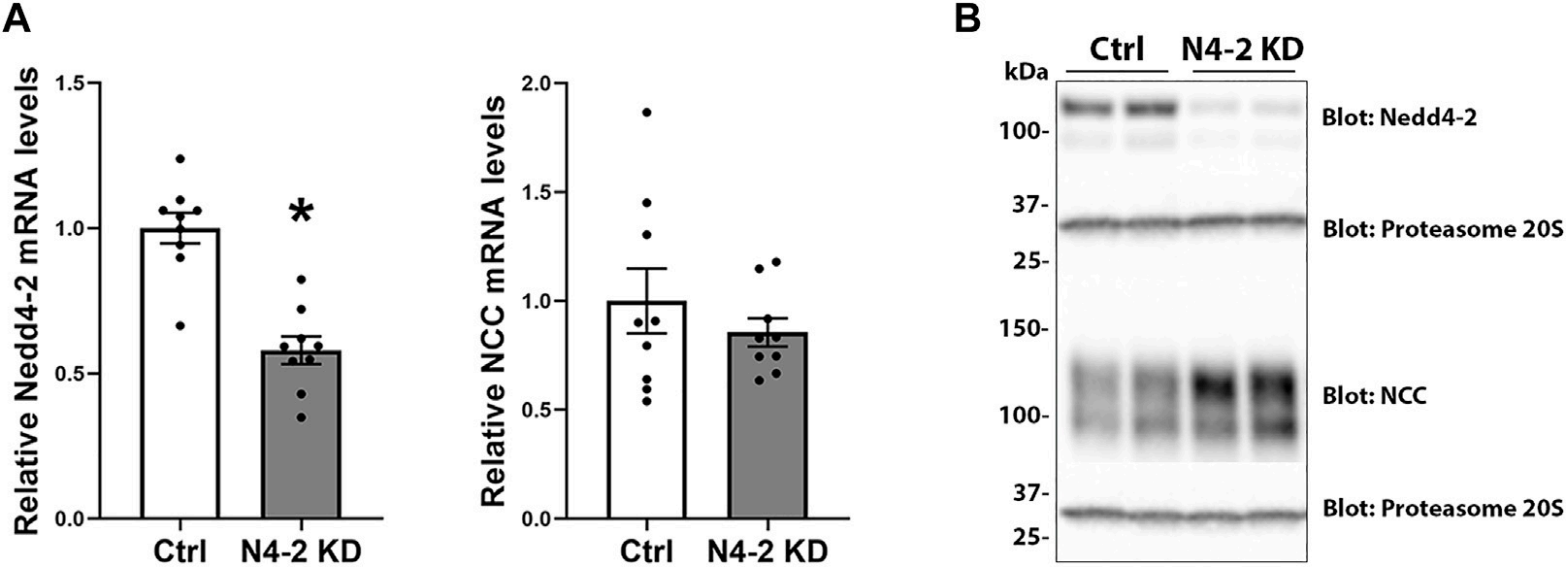
Nedd4-2 mRNA and protein levels in MDCKI-hNCC Nedd4-2 knockdown (N4-2 KD) cell lines. **(A)** mRNA levels of Nedd4-2 (left) and NCC (right) in MDCKI-hNCC Ctrl and N4-2 KD cell lines. The relative mRNA abundances are calculated from the Ct values using the delta-delta Ct method. Data are means ± S.E.M. (*n* = 9) *p < 0.05 assessed by unpaired Student’s t test. **(B)** Representative immunoblots of Nedd4-2, NCC, and proteasome 20S in MDCKI-hNCC Ctrl and N4-2 KD cell lines.

**FIGURE 3:**
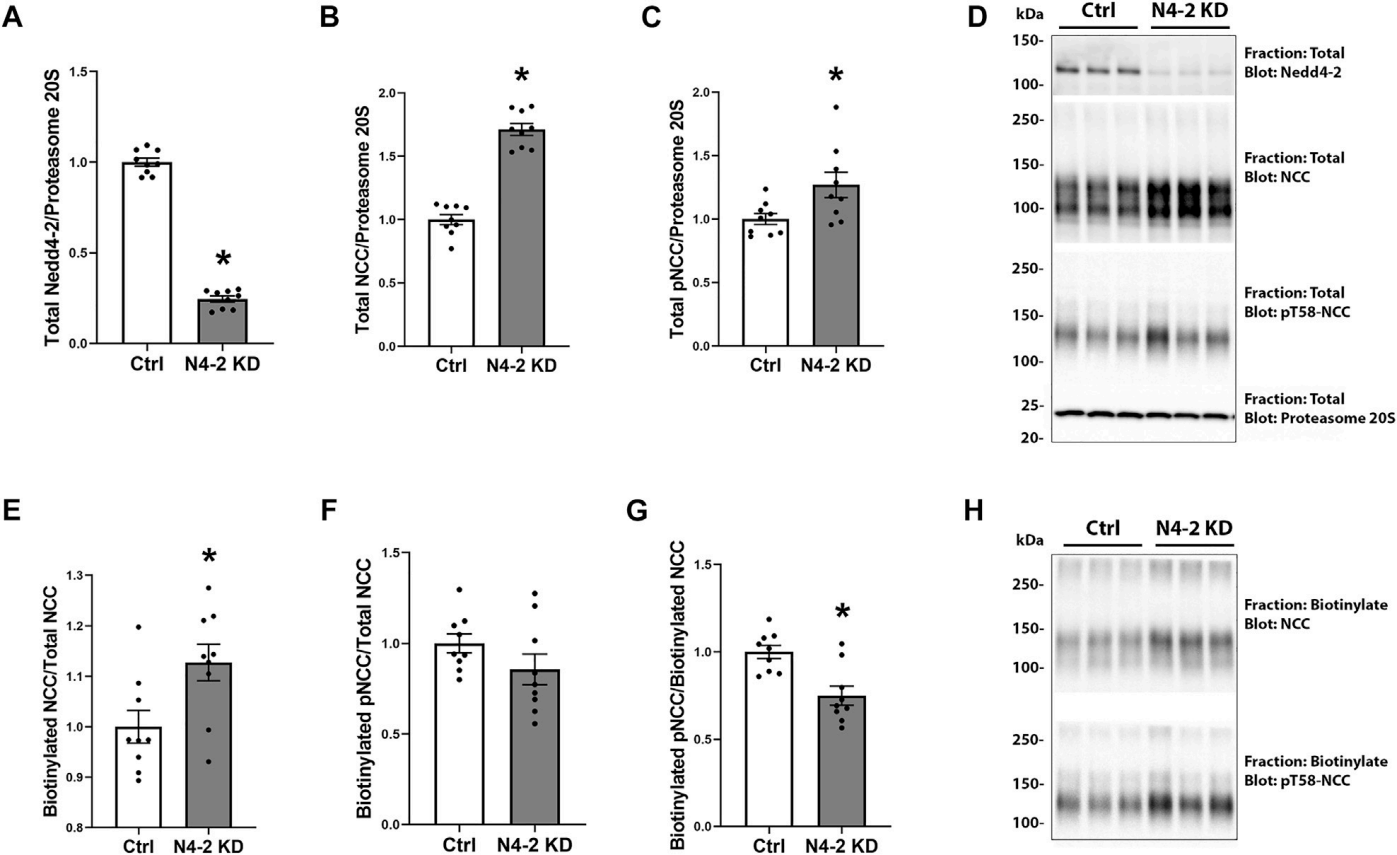
Nedd4-2 deficiency increases total NCC levels and plasma membrane abundance. Semi-quantitative assessment of Nedd4-2 **(A)**, NCC **(B)**, and pT58-NCC **(C)** levels in total cell lysates of MDCKI-hNCC Ctrl and Nedd4-2 (N4-2) KD cell lines. Data are means ± S.E.M. (*n* = 9) *p < 0.05 assessed by unpaired Student’s t-test. **(D)** Representative blots of Nedd4-2, NCC, and pT58-NCC in total cell lysates of MDCKI-hNCC Ctrl and N4-2 KD cell lines. Semi-quantitative assessment of NCC **(E)**, pT58-NCC **(F)** and the NCC/pT58-NCC ratio **(G)** in membrane fractions of MDCKI-hNCC Ctrl and Nedd4-2 (N4-2) KD cell lines. Data are means ± S.E.M. (*n* = 9) *p < 0.05 assessed by unpaired Student’s t-test. **(H)** Representative blots of NCC and pT58-NCC in membrane fractions of MDCKI-hNCC Ctrl and N4-2 KD cell lines.

To investigate the role of Nedd4-2 in regulation of NCC, a shRNA construct (shRNA targeting the HECT domain) was used to generate stable knockdown of Nedd4-2 in a MDCKI-hNCC cell line (Rosenbaek et al., 2017b). Several cell lines were initially characterized based on Nedd4-2 knockdown efficiency, cell morphology, and cell growth characteristics. Two of these cell lines were mixed (termed MDCKI-hNCC N4-2 KD) and used for the remainder of this study. A control cell line was generated using non-mammalian scrambled shRNA (named MDCKI-hNCC Ctrl). In MDCKI-hNCC N4-2 KD cells the levels of Nedd4-2 mRNA, assessed using RT-qPCR, were reduced to 58±4.7% (Figure 2A). No significant difference in NCC mRNA expression was observed between MDCKI-hNCC Ctrl and N4-2 KD cells (Figure 2A). In protein homogenates from MDCKI-hNCC Ctrl cells, Nedd4-2 was detected as a strong protein entity at approximately 120 kDa and a weaker band at 100 kDa (Figure 2B). In homogenates from MDCKI-hNCC N4-2 KD cells the intensities of these bands were reduced by approximately 75%, confirming successful knockdown of Nedd4-2 (Quantification from Figure 3A; 75.52±1.68%)

### Nedd4-2 deficiency increases total NCC levels and plasma membrane abundance

**FIGURE 4:**
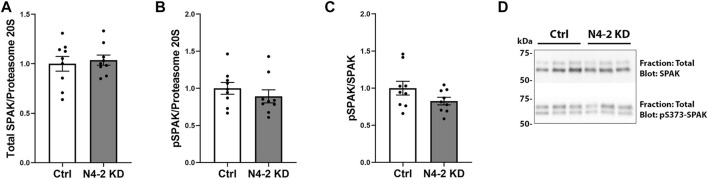
No significant difference in SPAK and phosphorylated SPAK (pS373-SPAK) are found in MDCKI-hNCC cells with Nedd4-2 KD. Semi-quantitative assessment of SPAK **(A)**, pS373-SPAK **(B)**, and pS373-SPAK/SPAK ratio **(C)** in total cell lysates of MDCKI-hNCC Ctrl and Nedd4-2 (N4-2) KD cell lines. Data are means ± S.E.M. (*n* = 9) *p < 0.05 assessed by unpaired Student’s t-test. **(D)** Representative blots of SPAK and pS373-SPAK in total cell lysates of MDCKI-hNCC Ctrl and N4-2 KD cell lines.

In total protein lysates from MDCKI-hNCC N4-2 KD cells the abundance of total and phosphorylated NCC were increased compared to control, corresponding with a significant decrease in Nedd4-2 levels (Figures 3A–D). To address if plasma membrane levels of NCC were altered after Nedd4-2 KD, MDCKI-hNCC N4-2 KD and Ctrl cells were biotinylated at the apical surface and the pool of membrane associated NCC relative to the total pool of NCC determined by western blotting. In the absence of Nedd4-2, the fraction of NCC in the membrane was significantly higher compared to MDCKI-hNCC Ctrl cells (Figures 3E,H). Phosphorylation levels of NCC at Threonine 58 (T58), an essential site for NCC function, were not significantly different at the plasma membrane (Figures 3F,H), although the fraction of phosphorylated relative to total NCC in the plasma membrane were significantly decreased (Figures 3G,H). The effects on NCC abundance and plasma membrane levels appear to be independent of the WNK-SPAK pathway, with no significant differences in total or phosphorylated SPAK in MDCKI-hNCC N4-2 KD cells compared to control cells (Figures 4A–D).

### Nedd4-2 deficiency reduces total and membrane NCC ubiquitylation, increases NCC protein half-life but does not delay internalization of NCC

**FIGURE 5:**
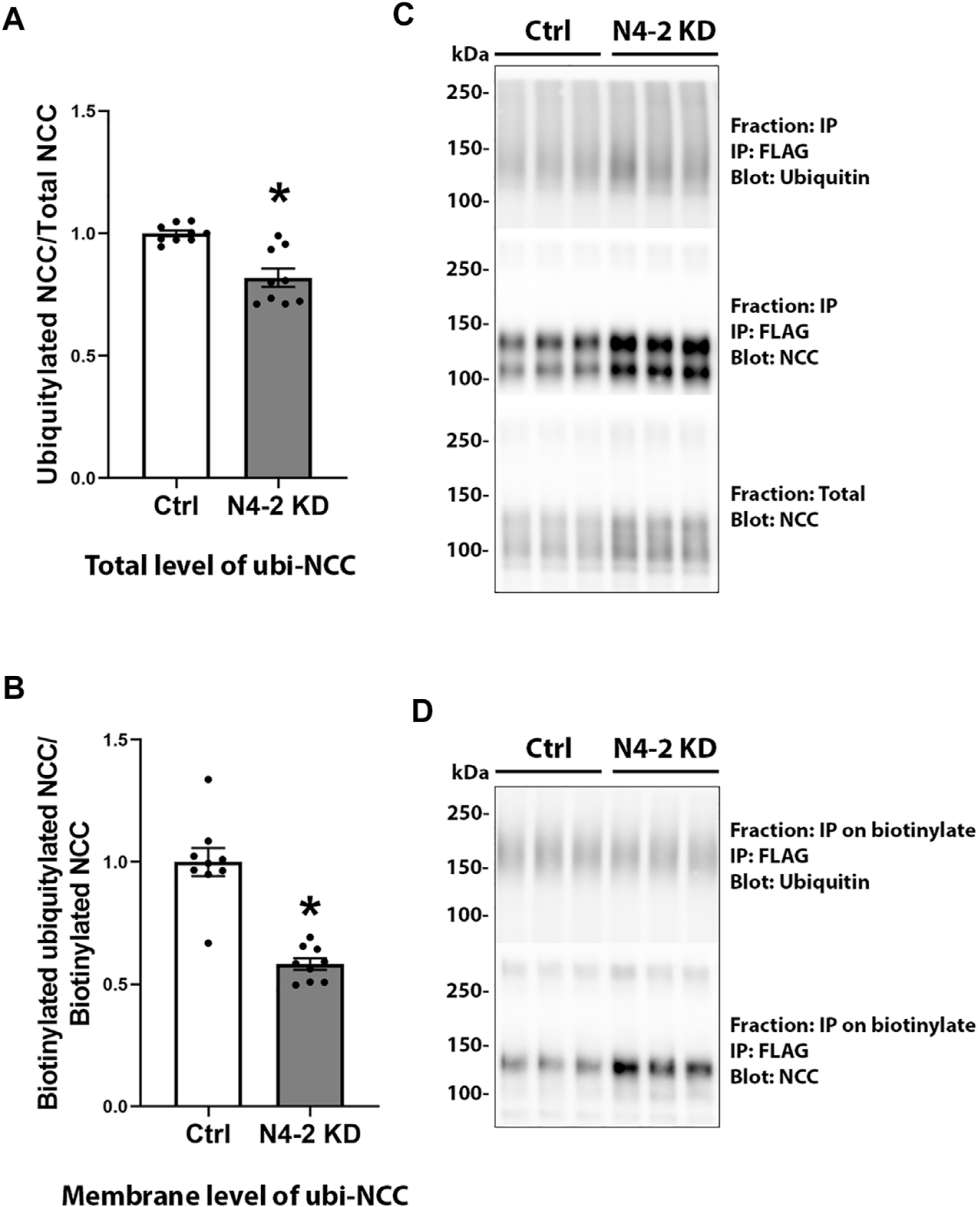
Ubiquitylation levels of NCC in total lysates and membrane fraction are decreased after Nedd4-2 knock down. Semi-quantitative assessment of ubiquitylation levels of NCC in total cell lysates **(A)** and membrane fractions **(B)** of MDCKI-hNCC Ctrl and Nedd4-2 (N4-2) KD cell lines. Data are means ± S.E.M. (n = 9) *p < 0.05 assessed by unpaired Student’s t-test. Representative immunoblots of ubiquitylated NCC and total NCC from total cell lysates are shown in **(C)** and the membrane fraction in **(D)**.

**FIGURE 6:**
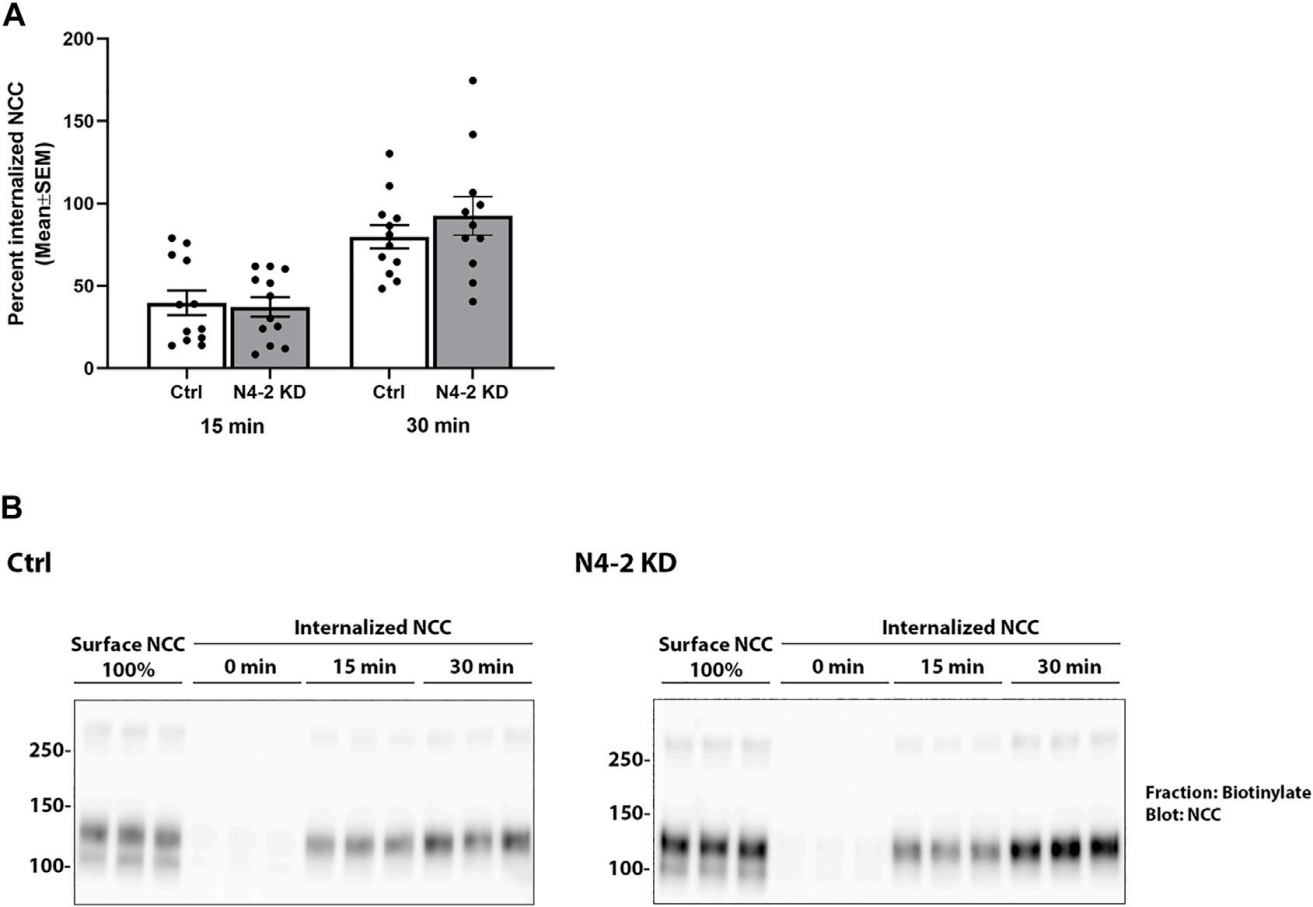
Nedd4-2 deficiency does not change the constitutive internalization of NCC from the apical plasma membrane in MDCKI-hNCC cells. **(A)** Semi-quantitative assessment of the percentage of internalized NCC (steady-state surface levels equals 100%) at 15 and 30 min. Data are means ± S.E.M. (*n* = 12). Significance is assessed with 2way ANOVA followed by Tukey’s multiple comparisons test. **(B)** Representative blots showing NCC internalization. Following biotinylation, MDCKI-hNCC Ctrl and Nedd4-2 (N4-2) KD cells were incubated for 15 or 30 min at 37˚C to allow membrane protein internalization before treatment with the reducing agent MesNa (stripping biotin). Lane 1-3 show expression of steady-state surface NCC. Lane 4-6 show surface NCC after treatment with the stripping agent MesNa. Internalized NCC was isolated, detected by western blot, and quantified by densitometry.

**FIGURE 7:**
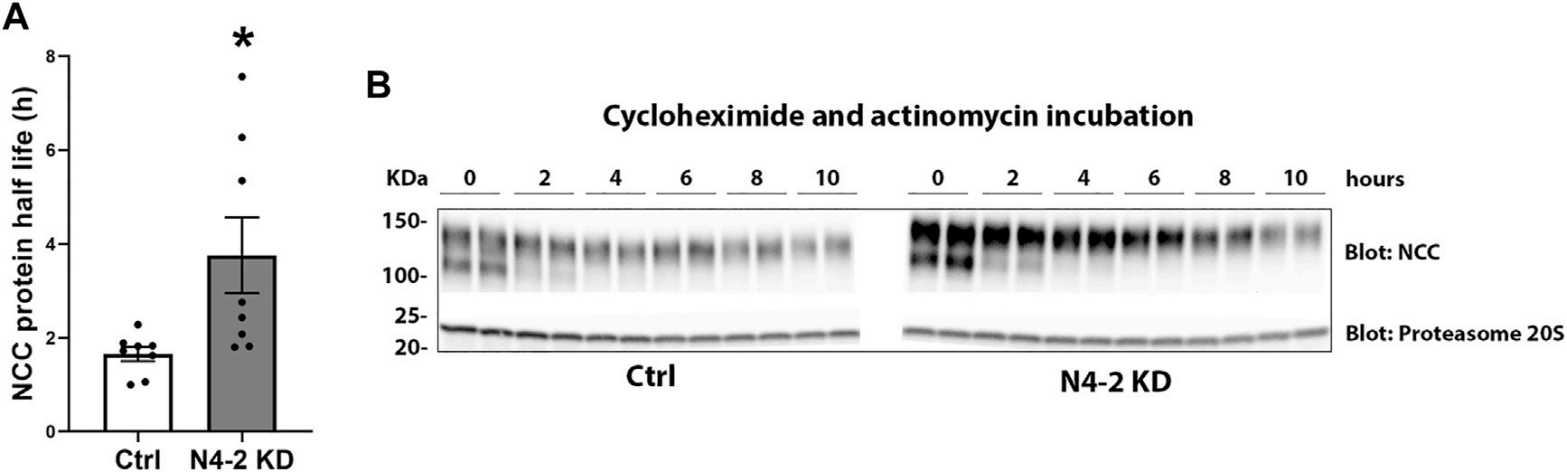
NCC half-life is increased with Nedd4-2 deficiency in MDCKI-hNCC cells. **(A)** Semi-quantitative assessment of NCC protein half-life from 8 independent experiments. Data are means ± S.E.M. *p < 0.05 assessed by unpaired Student’s t-test. **(B)** Representative immunoblots of NCC in cell lysates isolated from MDCKI-hNCC Ctrl and Nedd4-2 (N4-2) KD cells treated with cycloheximide and actinomycin for the indicated timepoints. Half-lives were calculated for each experiment with a one-phase exponential decay analysis.

Knockdown of Nedd4-2 significantly decreased the fraction of total cellular NCC that was ubiquitylated to 81.89±3.8% of the levels observed in MDCKI-hNCC Ctrl cells (Figures 5A,C). Furthermore, the fraction of ubiquitylated NCC relative to total NCC at the plasma membrane was significantly decreased in MDCKI-hNCC N4-2 KD cells compared to control (Figures 5B,D). We have previously shown site-specific ubiquitylation to be involved in internalization of NCC (Rosenbaek et al., 2017a). Therefore, constitutive NCC internalization was investigated using biotin-based endocytosis-assays. However, at the time points examined no significant difference was detected in the degree of internalization of NCC from the plasma membrane in MDCKI-hNCC N4-2 KD cells compared to Ctrl cells (Figures 6A,B). The increase in NCC protein abundance in the absence of Nedd4-2 without alterations in NCC mRNA levels suggest altered ubiquitin-mediated NCC degradation (Rosenbaek et al., 2017a). To confirm this, NCC protein chase studies were performed after inhibition of RNA transcription and protein translation. In MDCKI-hNCC N4-2 KD cells NCC half-life was calculated to be 3.76 ± 0.81 h, which is significantly longer than the 1.655 ± 0.15 h observed in MDCKI-hNCC Ctrl cells, suggesting a slower rate of NCC degradation after Nedd4-2 KD (Figures 7A,B).

### Increased extracellular K^+^ reduces NCC abundance even in the absence of Nedd4-2

**FIGURE 8:**
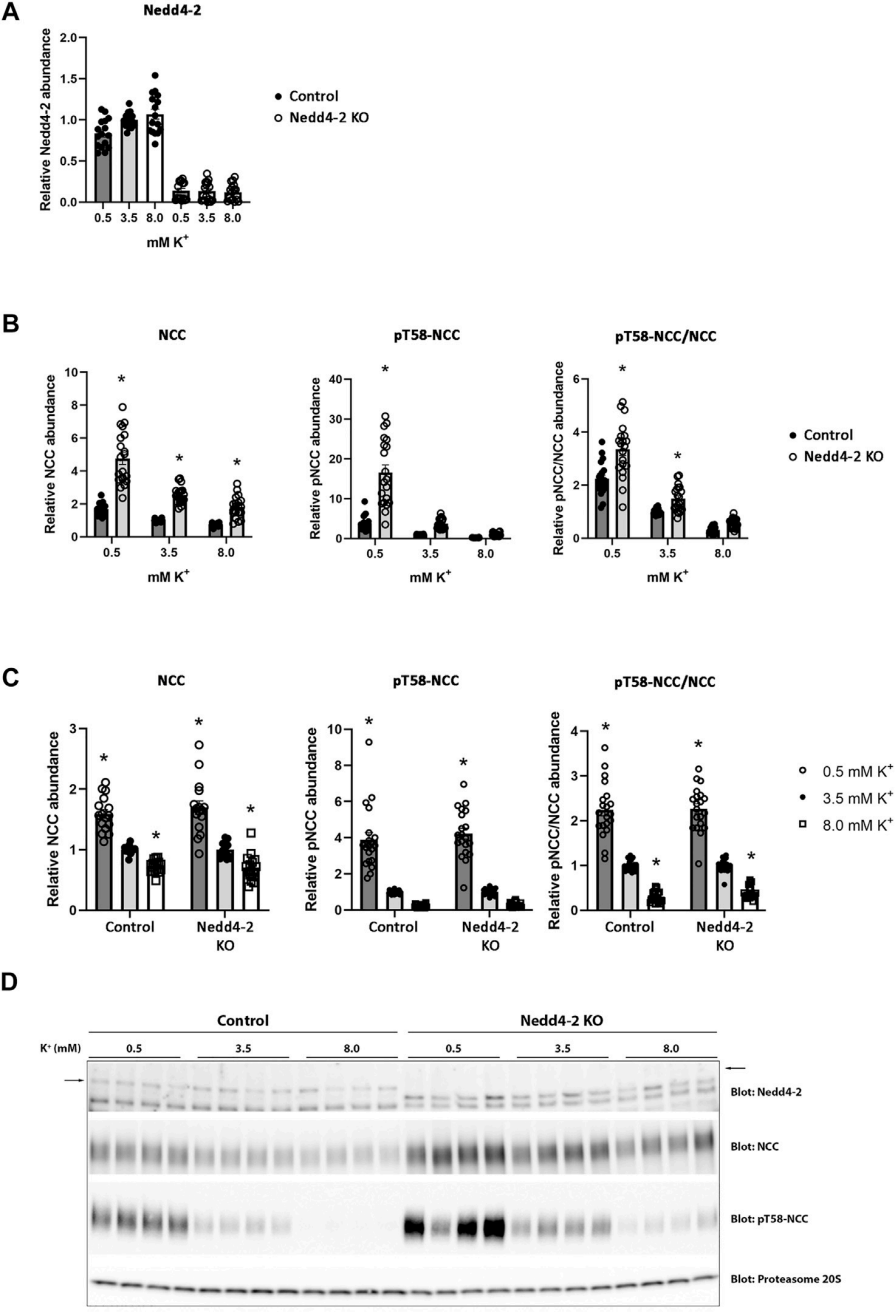
Nedd4-2 is not involved in the potassium induced regulation of NCC and pT58-NCC. Semi-quantitative assessment of Nedd4-2 **(A)**, NCC, pT58-NCC, and pT58-NCC/NCC **(B+C)** levels in tubules isolated from control and Nedd4-2 KO mice and treated with 0.5 mM, 3.5 mM, or 8.0 mM K^+^ for 24 h. **(A+B)** Data are means ± S.E.M and normalized to control tubules treated with 3.5 mM K^+^. Difference between control and KO tubules treated with either 0.5 mM, 3.5 mM, or 8.0 mM K^+^ is assessed by 2way ANOVA followed by Tukey’s multiple comparisons test (*p < 0.05). **(C)** Data are means ± S.E.M. Control and KO tubules are normalized to their own 3.5 mM K^+^ group. Differences of NCC and pT58-NCC levels with different K^+^ concentrations in either control or KO tubules are assessed by 2way ANOVA followed by Tukey’s multiple comparisons test (*p < 0.05). Fold changes between control and KO tubules with changing K^+^ concentrations are assessed in the same 2way ANOVA analysis (n = 16–20). **(D)** Representative immunoblots of Nedd4-2, NCC, pT58-NCC, and proteasome 20S in control and KO tubules treated with 0.5 mM, 3.5 mM, or 8.0 mM K^+^.

**FIGURE 9:**
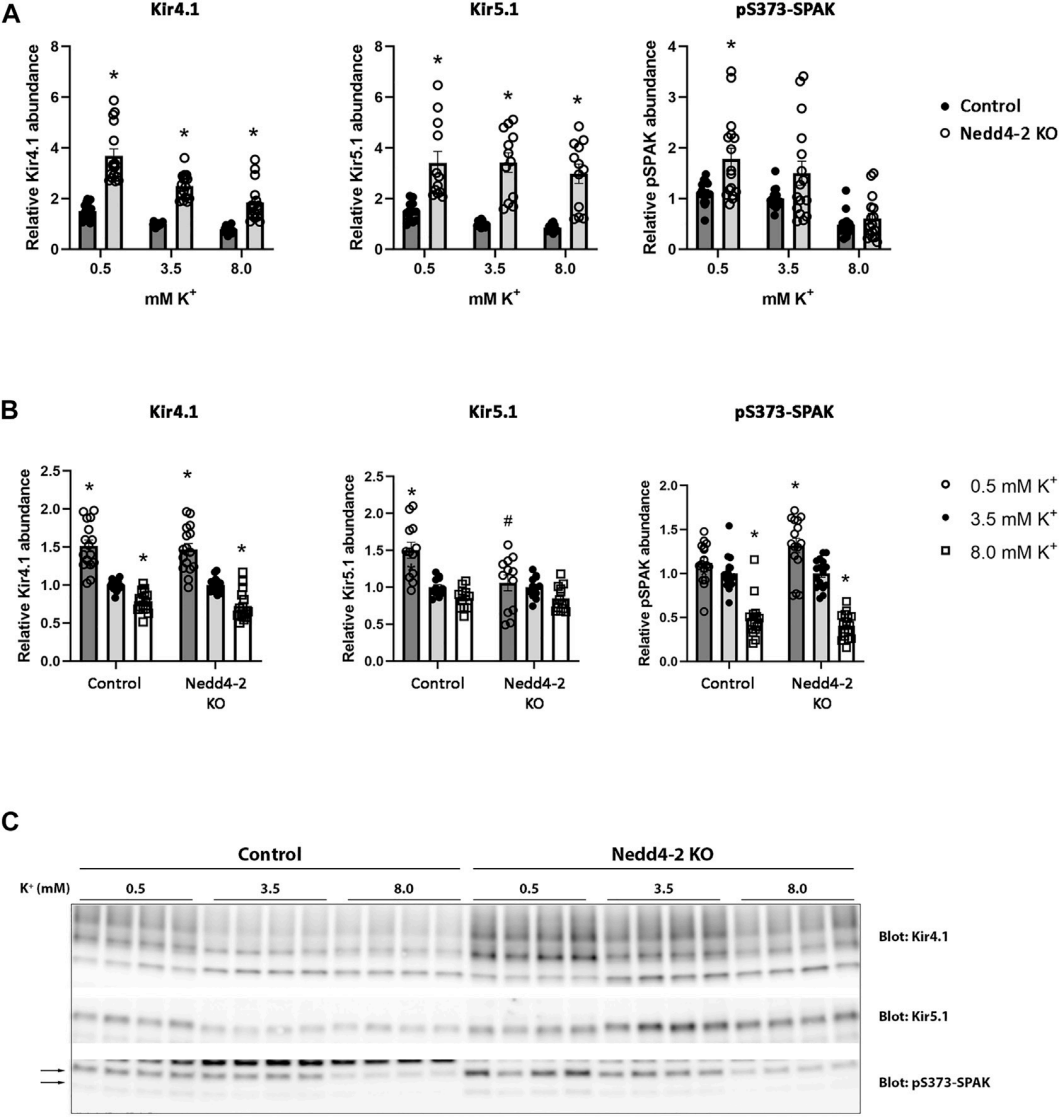
Kir4.1, Kir5.1, and pS373-SPAK levels are increased in tubule suspensions isolated from Nedd4-2 KO mice.**(A+B)** Semi-quantitative assessment of Kir4.1, Kir5.1, and pS373-SPAK levels in tubules isolated from control and Nedd4-2 KO mice treated with 0.5 mM, 3.5 mM, or 8.0 mM K^+^ for 24 h. **(A)** Data are means ± S.E.M and normalized to control tubules treated with 3.5 mM K^+^. Difference between control and KO tubules treated with either 0.5 mM, 3.5 mM, or 8.0 mM K^+^ is assessed by 2way ANOVA followed by Tukey’s multiple comparisons test (*p < 0.05). **(B)** Data are means ± S.E.M. Control and KO tubules are normalized to their own 3.5 mM K^+^ group. Differences of Kir4.1, Kir5.1, and pS373-SPAK levels with different K^+^ concentrations in either control or KO tubules are assessed by 2way ANOVA followed by Tukey’s multiple comparisons test (*p < 0.05). Fold changes between control and KO tubules with changing K^+^ concentrations are assessed in the same 2way ANOVA analysis (^#^ *p* < 0.05) (n = 12–16). **(C)** Representative immunoblots of Kir4.1, Kir5.1, and pS373-SPAK in control and KO tubules treated with 0.5 mM, 3.5 mM, or 8.0 mM K^+^.

The abundance of NCC is reduced by high extracellular K^+^ concentrations (Frindt and Palmer, 2010; Sorensen et al., 2013; van der Lubbe et al., 2013; Rengarajan et al., 2014; Terker et al., 2015). The mechanism/s responsible for this are unclear, but increased NCC degradation plays a role (Kortenoeven et al., 2021). After determining that Nedd4-2 activity is important for modulating NCC abundance and degradation in MDCKI cells, we next tested if Nedd4-2 was important for lowering NCC abundance in response to high extracellular K^+^. As long-term effects on NCC are technically difficult to study in the inducible MDCKI-hNCC cell system, we switched to using *ex vivo* kidney tubule preparations (Cheng et al., 2019; Kortenoeven et al., 2021; Murali et al., 2021; Poulsen et al., 2021) from mice lacking Nedd4-2 in the kidney tubule (referred to as Nedd4-2 KO) and relevant control mice. Tubules were incubated with different concentrations of K^+^ (0.5, 3.5, and 8 mM) for 24 h and protein abundances were assessed using western blotting. Nedd4-2 abundance was significantly lower in tubules isolated from Nedd4-2 KO mice compared to the tubules from control mice, but no effects of altered extracellular K^+^ on Nedd4-2 were observed (Figures 8A,D). In tubules isolated from Nedd4-2 KO mice, the abundance of NCC was significantly higher compared to tubules from control mice. Phosphorylated NCC was also significantly higher in Nedd4-2 KO tubules after stimulation with low K^+^, as was the ratio of pT58-NCC to NCC, except when tubules were treated with high K^+^ (Figures 8B,D). The fold change of NCC and pT58-NCC subsequent to alterations in extracellular K^+^ were assessed by normalizing control or Nedd4-2 KO tubules to their own 3.5 mM K^+^. The responses of NCC and pT58-NCC to low or high K^+^ were similar in tubules from control and Nedd4-2 KO tubules and not significantly different from each other (Figure 8C). Kidney-specific deletion of Nedd4-2 increases expression and activity of the basolateral potassium (K^+^) channel Kir4.1 *in vivo* resulting in activation of the WNK-SPAK pathway (Wu et al., 2020). In line with this, Kir4.1 and Kir5.1 protein abundances were greater in tubules from Nedd4-2 KO mice compared to controls, but the changes in abundances in response to altered extracellular K^+^ were similar. Fitting with the higher abundances of Kir4.1/5.1, pS373-SPAK (active SPAK) levels on average were higher in tubules from Nedd4-2 KO mice (Figure 9A+C). Furthermore, low K^+^ increased pS373-SPAK in Nedd4-2 KO tubules greater than controls, although the effects of high K^+^ were independent of genotype (Figure 9B).

## Discussion

NCC activity is altered by phosphorylation, and an indirect role for the E3 ubiquitin protein ligase KLHL3-CUL3 complex to alter NCC phosphorylation via the WNK-SPAK pathway is well established. Direct multisite ubiquitylation of NCC is also important for its regulation (Rosenbaek et al., 2017a), and data supporting a role for the E3 ubiquitin protein ligase Nedd4-2 is slowly evolving. However, the mechanisms by which Nedd4-2 modulates NCC and whether Nedd4-2 plays a role in mediating K^+^ inhibitory effects on NCC were unclear. Here, we provide substantial evidence that Nedd4-2 most likely directly interacts with and ubiquitylates NCC, the consequence of which is both reduced NCC membrane abundance and increased NCC degradation. Despite this clear regulatory role of Nedd4-2, we also demonstrate that Nedd4-2 activity is not critically important for the reduction of NCC phosphorylation and abundance that occurs with short-term increased extracellular K^+^.

Y2H assays identified three E3 protein ligases that interacted with the C-terminus of mouse NCC; Nedd4-2, Nedd4, and the RING finger type E3 ligase Pdzrn3. The role of Nedd4 and Pdzrn3 for regulating NCC is the basis of ongoing studies, whereas this study focused on Nedd4-2 modulation of NCC. Although Nedd4-2 and NCC have previously been shown to co-immunoprecipitate (Arroyo et al., 2011), the Y2H assays suggest that their interaction may be direct and occurs within a 216 amino acid stretch of the N-terminus of Nedd4-2 (Figure 1A). However, Nedd4-2 usually interacts through binding of its WW domains to PPxY (PY) or with lesser tendency to LPxY motifs in substrates and regulatory proteins, neither of which are present in NCC. A PY-like motif (843 … TLLIPYLLGR … 852) does exist in the C-terminus of NCC but mutating this site (NCC-P847A) does not prevent NCC-Nedd4-2 immunoprecipitation, suggesting they interact independently of this motif (Arroyo et al., 2011). It is plausible that Nedd4-2 and NCC interact through non-PY/LPxY motifs, for example via phosphorylated tyrosine- or serine residues, a mechanism that accounts for 40% of the human Nedd4-2 interactome (Hatstat et al., 2021). However, unlike the NH2-terminus, the C-terminus of NCC is not heavily phosphorylated and therefore how the interaction between NCC and Nedd4-2 occurs remains unresolved.

A stable, approximately 75%, knock down of Nedd4-2 in NCC expressing MDCKI cells (derived from dog) was achieved by targeting the HECT domain (exon 22–31) of Nedd4-2 with shRNA. At the start of this study the dog Nedd4-2 gene contained 38 exons encoding 10 different mRNA isoforms (NCBI database). Now 16 different isoforms of dog Nedd4-2 are predicted, so it is possible that the remaining Nedd4-2 protein corresponds to the other, or even unknown isoforms. Total Nedd4-2 KO and inducible renal tubule-specific Nedd4-2 KO mice have been generated deleting exons 6 to 8 of the *Nedd4L* gene (Shi et al., 2008; Ronzaud et al., 2013). In our hands, shRNAs targeting upstream of the HECT domain (exon 17), did not result in a greater Nedd4-2 knock down efficiency (not shown). Whether a full knockout of Nedd4-2 in the NCC expressing MDCKI cell line could be achieved using CRISPR/Cas9 technology is unclear, but it is unlikely to yield results greatly different to what we have uncovered using the shRNA approach.

The decreased ubiquitylation levels of total and plasma membrane NCC with Nedd4-2 deficiency strengthens the idea that Nedd4-2 ubiquitylates NCC. One can speculate whether the remaining ubiquitylation of NCC is caused by the “leftover” Nedd4-2. Alternatively, as NCC is heavily ubiquitylated on at least 12 lysine residues, it is possible that various E3 ligases including Nedd4 and Pdzrn3 are responsible for ubiquitylation at different sites. These ligases may also compensate partially when Nedd4-2 activity is removed, although preliminary experiments suggest that Nedd4 mRNA expression (Supplementary Figure S1) in MDCKI-hNCC N4-2 KD cells does not differ from controls (Pdzrn3 was not detectable in MDCK cells). Furthermore, although an inverse relationship exists between NCC phosphorylation and ubiquitylation levels at the plasma membrane (Khan et al., 2012; Rosenbaek et al., 2014), we did not observe altered NCC phosphorylation at the plasma membrane when NCC ubiquitylation levels were decreased, indicating that the Nedd4-2 induced ubiquitylation does not interfere with NCC phosphorylation. When considering the large number of ubiquitylation sites in NCC relative to phosphorylation sites, it is highly probable that only some ubiquitylation sites are involved in the opposing effects on plasma membrane levels.

Dependent on the type of ubiquitylation, for example whether it is mono- or polyubiquitylation or the type of ubiquitin linkage and branching, the effects on the targeted protein can vary from altered endocytosis or protein trafficking, to enhanced proteasomal or lysosomal degradation (Akutsu et al., 2016). The increased levels of both total and plasma membrane NCC (relative to total) following Nedd4-2 knockdown supports the idea that Nedd4-2 induced ubiquitylation targets NCC for degradation, but also alters NCC trafficking. As we detected no differences in the internalization rate of NCC with Nedd4-2 deficiency, the observed differences in membrane abundance are not due to decreased NCC endocytosis, indicating increased NCC exocytosis. Earlier studies suggested that activation of the WNK4-SPAK pathway and NCC phosphorylation can promote NCC exocytosis (Cai et al., 2006; Subramanya et al., 2009), but other studies have indicated that NCC phosphorylation occurs directly in the plasma membrane (Pedersen et al., 2010; Rosenbaek et al., 2012). Furthermore, we observed no differences in the abundance or phosphorylation of SPAK, so it is unlikely that there is enhanced activation of the WNK-SPAK pathway in MDCKI-hNCC cells in the absence of Nedd4-2, and the basis of the increased NCC exocytosis remains elusive.

Recently, it was demonstrated that high extracellular K^+^ enhances ubiquitin-dependent degradation of NCC (Kortenoeven et al., 2021). In the current study, in the absence of Nedd4-2 the abundance of NCC was increased and NCC protein half-life increased, suggesting a critical role of Nedd4-2 in NCC degradation. Therefore, we hypothesized that Nedd4-2 was also involved in high K^+^ driven NCC degradation. Confirming our data in MDCKI-NCC cells, NCC levels were higher in *ex vivo* kidney tubules from Nedd4-2 KO mice relative to tubules from control mice. However, we found no significant differences in the ability of high K^+^ to reduce NCC abundance and phosphorylation in the absence of Nedd4-2. Similarly, an earlier study by Al-Qusairi *et al.* (Al-Qusairi et al., 2017) demonstrated daily kaliuresis in renal tubule Nedd4-2 deficient mice fed a high K^+^ diet for 5 days was similar to control mice, suggesting that NCC can be suppressed *in vivo* despite Nedd4-2 deletion. The current and previous data (Al-Qusairi et al., 2017) are in contrast to a recent *in vivo* study by Xiao *et al.*, where high dietary K^+^ effects on NCC and phosphorylated NCC (at Thr53) were diminished with Nedd4-2 deletion (Xiao et al., 2021). The reasons for these differences are unclear, but it could be related to time. Xiao *et al.* examined NCC abundance after mice were fed a high K^+^ diet (5% K^+^ and 0.4% Na^+^) for 7 days, whereas our *ex vivo* tubules were treated with 8.0 mM extracellular K^+^ for only 24 h and the *in vivo* study by Al-Qusairi *et al.* was for 5 days (Al-Qusairi et al., 2017). Thus, Nedd4-2 may be dispensable for earlier K^+^-induced effects on NCC (no effects observed in tubules or short-term dietary K^+^ feeding), but essential for a sustained ability of high K^+^ to reduce NCC. In line with this idea, as seen *in vivo* (Wang et al., 2018a; Wu et al., 2020), Kir4.1 and Kir5.1 abundances were higher in Nedd4-2 KO tubules, but the decrease in Kir4.1 was still observed in the absence of Nedd4-2. Interestingly the increase in Kir5.1 with low K^+^ was not seen in the Nedd4-2 KO tubules, confirming Nedd4-2 to act on Kir5.1 rather than Kir4.1 (Wang et al., 2018b). Of caution, in this study we examined the expression of Kir4.1 and Kir5.1 in the absence of Nedd4-2 and we cannot rule out changes in the activity of the Kir4.1/kir5.1 heterotetramer, as changes in the basolateral K^+^ conductance in the DCT are not always linked to an increase in the expression of Kir4.1 (Wang et al., 2018a). Previous studies also support the idea that different mechanisms are responsible for reducing NCC phosphorylation and abundance in a time-dependent manner. For example, in a mouse model with sustained activation of the WNK-SPAK pathway, the reduction in NCC abundance and phosphorylation subsequent to acute K^+^ loading is attenuated (Chen et al., 2019). However, similar reductions in NCC phosphorylation and abundance are seen in this mouse model relative to control mice after 4 days of a high K^+^ diet, suggesting that WNK-SPAK independent mechanisms are occurring during this period. Furthermore, acute K^+^ loading only decreased NCC phosphorylation in mice that were prior K^+^-restricted for 1 day, but not in mice that were chronically K^+^-restricted for 5 days (Mukherjee et al., 2021).

In conclusion, our *in vitro* and *in vivo* studies strongly support the concept that Nedd4-2 is an important regulator of cellular NCC abundance and plasma membrane expression, likely driven by its ability to increase NCC degradation and enhance NCC exocytosis, respectively. However, our data do not support an initial direct role of Nedd4-2 in K^+^ induced degradation of NCC.

## Data Availability

The original contributions presented in the study are included in the article/Supplementary Material, further inquiries can be directed to the corresponding author.
