# Supplementary material for: The E3 ubiquitin-protein ligase Nedd4-2 regulates the sodium chloride cotransporter NCC but is not required for a potassium-induced reduction of NCC expression
Source: Front Physiol. 2022 Sep 7;13:971251. doi: 10.3389/fphys.2022.971251 (PMC9490057; doi:10.3389/fphys.2022.971251)
Supplement: Supplementary file 1 [file Table1.PDF]

Gene name	Annotation	SID
**Ankrd44**	[gi|304376296|ref|NM_001081433.3| Mus musculus ankyrin repeat domain 44 (Ankrd44), mRNA]	365-732
**Kif13b**	[gi|124487162|ref|NM_001081177.1| Mus musculus kinesin family member 13B (Kif13b), mRNA]	1396-1459
**Lage3**	[NM_025410.2 Mus musculus L antigen family, member 3 (Lage3), mRNA]	10-148
**Lap3**	[gi|255069714|ref|NM_024434.6| Mus musculus leucine aminopeptidase 3 (Lap3), mRNA]	16-331
**Mast2**	[gi|545688905|ref|NM_001042743.2| Mus musculus microtubule associated serine/threonine kinase 2 (Mast2), transcript variant 1, mRNA]	965-1319
**Nav1**	[gi|224922750|ref|NM_173437.2| Mus musculus neuron navigator 1 (Nav1), mRNA]	1285-1499
**Nedd4**	[gi|56699422|ref|NM_010890.3| Mus musculus neural precursor cell expressed, developmentally down-regulated gene 4 (Nedd4), mRNA]	124-430
**Nedd4l**	[gi|167466244|ref|NM_001114386.1| Mus musculus neural precursor cell expressed, developmentally down-regulated gene 4-like (Nedd4l), transcript variant 1, mRNA]	1-216
**Numb**	[XM_006515575.2 PREDICTED: Mus musculus numb homolog (Drosophila) (Numb), transcript variant X4, mRNA]	13-575
**Pdzrn3**	[gi|256985135|ref|NM_018884.2| Mus musculus PDZ domain containing RING finger 3 (Pdzrn3), mRNA]	803-1015
**Ranbp10**	[gi|117676379|ref|NM_145824.4| Mus musculus RAN binding protein 10 (Ranbp10), mRNA]	26-433
**Senp3**	[gi|254939658|ref|NM_030702.4| Mus musculus SUMO/sentrin specific peptidase 3 (Senp3), transcript variant 1, mRNA]	184-568
**Sord**	[gi|158508518|ref|NM_146126.4| Mus musculus sorbitol dehydrogenase (Sord), mRNA]	N/A
**Tmem8**	[NM_021793.2 Mus musculus transmembrane protein 8 (five membrane-spanning domains) (Tmem8), mRNA]	1-43
**Wwp1**	[gi|443906718|ref|NM_177327.5| Mus musculus WW domain containing E3 ubiquitin protein ligase 1 (Wwp1), transcript variant 1, mRNA]	52-366
**Zbtb16**	[gi|142382613|ref|NM_001033324.2| Mus musculus zinc finger and BTB domain containing 16 (Zbtb16), mRNA]	423-673

Supplemental Table 1: Yeast two hybrid results
