# Supplementary figures and images for: The E3 ubiquitin-protein ligase Nedd4-2 regulates the sodium chloride cotransporter NCC but is not required for a potassium-induced reduction of NCC expression
Source: Front Physiol. 2022 Sep 7;13:971251. doi: 10.3389/fphys.2022.971251 (PMC9490057; doi:10.3389/fphys.2022.971251)

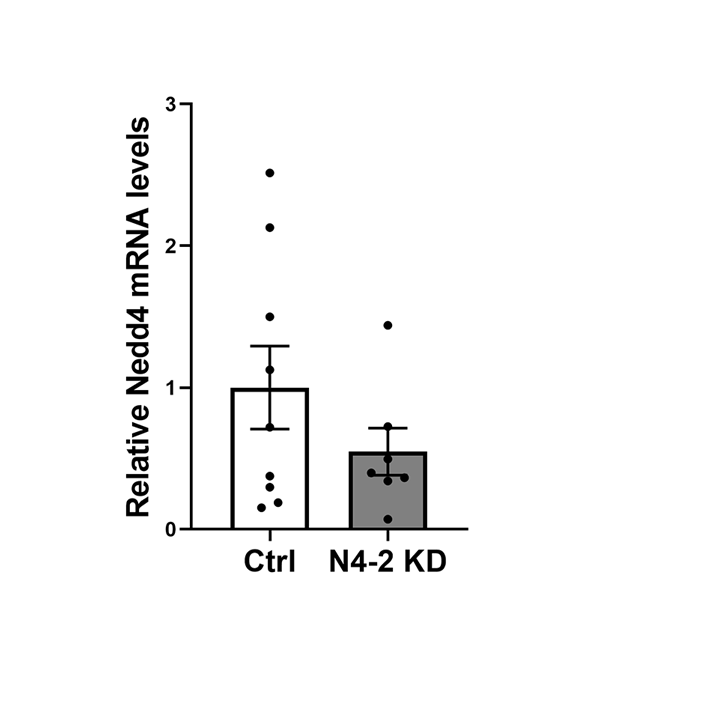

Supplement: Supplementary file 2 [file Image1.TIF]
